# GrTCP11, a Cotton TCP Transcription Factor, Inhibits Root Hair Elongation by Down-Regulating Jasmonic Acid Pathway in *Arabidopsis thaliana*

**DOI:** 10.3389/fpls.2021.769675

**Published:** 2021-11-22

**Authors:** Juan Hao, Panpan Lou, Yidie Han, Zhehao Chen, Jianmei Chen, Jun Ni, Yanjun Yang, Zhifang Jiang, Maojun Xu

**Affiliations:** Zhejiang Provincial Key Laboratory for Genetic Improvement and Quality Control of Medicinal Plants, Hangzhou Normal University, Hangzhou, China

**Keywords:** TCP transcription factor, *Gossypium raimondii*, fiber, *Arabidopsis thaliana*, root hair, jasmonic acid

## Abstract

TCP transcription factors play important roles in diverse aspects of plant development as transcriptional activators or repressors. However, the functional mechanisms of TCPs are not well understood, especially in cotton fibers. Here, we identified a total of 37 non-redundant TCP proteins from the diploid cotton (*Gossypium raimondii*), which showed great diversity in the expression profile. GrTCP11, an ortholog of AtTCP11, was preferentially expressed in cotton anthers and during fiber initiation and secondary cell wall synthesis stages. Overexpression of *GrTCP11* in *Arabidopsis thaliana* reduced root hair length and delayed flowering. It was found that *GrTCP11* negatively regulated genes involved in jasmonic acid (JA) biosynthesis and response, such as *AtLOX4*, *AtAOS*, *AtAOC1*, *AtAOC3*, *AtJAZ1*, *AtJAZ2*, *AtMYC2*, and *AtERF1*, which resulted in a decrease in JA concentration in the overexpressed transgenic lines. As with the JA-deficient mutant *dde2-2*, the transgenic line 4-1 was insensitive to 50 μM methyl jasmonate, compared with the wild-type plants. The results suggest that GrTCP11 may be an important transcription factor for cotton fiber development, by negatively regulating JA biosynthesis and response.

## Introduction

TCP transcription factors are plant-specific developmental regulators, named according to the first four characterized members of the family, namely maize (*Zea mays*) TEOSINTE BRANCHED1, snapdragon (*Antirrhinum majus*) CYCLOIDEA, and rice (*Oryza sativa*) PROLIFERATING CELL NUCLEAR ANTIGEN FACTOR1 and 2. The main feature of TCP proteins is a conserved non-canonical basic-Helix-Loop-Helix structure near the N-terminus, known as the TCP domain, which is consisting of approximately 60 amino-acid residues (Cubas et al., 1999). The TCP transcription family can be categorized into two subfamilies, namely class I and class II, based on sequence similarities of the TCP domain, which act as activators or repressors by the formation of homodimers or heterodimers (Kosugi and Ohashi, 2002). They have been shown to be involved in a wide range of biological processes throughout the entire life span of plants. One of the founding members of the TCP family, AmCYC, controls floral asymmetry (Luo et al., 1996). AtTCP14 and AtTCP15 redundantly modulate internode elongation and leaf shape in *Arabidopsis* (Kieffer et al., 2011). GhTCP12, GhTCP13, and GhTCP18 are functionally redundant in controlling branching in cotton and *Arabidopsis* (Diao et al., 2019). AtTCP2, AtTCP3, AtTCP11, and AtTCP15 were found to participate in the regulation of circadian clock by interacting with different components (Giraud et al., 2010). The expression of a repressor form of AtTCP11 resulted in smaller and curly leaves, shorter petioles, pedicels, and siliques, as well as a higher proportion of abnormal seeds and pollens. The presence of a threonine residue at position 15 of the TCP domain is responsible for the distinct DNA-binding properties of AtTCP11 (Viola et al., 2011). Knockdown of AsPCF5, AsPCF6, AsPCF8, and AsTCP14 by overexpression of miR319 increases tolerance to dehydration and salinity stress in creeping bentgrass (Zhou et al., 2013). However, the molecular mechanisms by which TCPs are involved in plant development are largely unknown.

Recent studies have revealed that many TCP proteins seem to play central roles in biological processes by regulating phytohormone signals. PsBRC1 plays a key role in integrating strigolactone and cytokinin signals in pea to control shoot branching (Braun et al., 2012). CIN limits excess cell proliferation and maintains the flatness of the leaf surface by directly regulating the expression of cytokinin- and auxin-related genes in *Antirrhinum majus* (Das Gupta et al., 2014). AtTCP14 regulates seed germination by affecting response to abscisic acid and gibberellin signals (Tatematsu et al., 2008). AtTCP15 modulates gynoecium development by influencing the balance between auxin and cytokinin levels (Lucero et al., 2015). AtTCP1 mediates plant growth and development by directly inducing the expression of *DWF4* and thus promoting brassinosteroid biosynthesis (Gao et al., 2015). Contrary to class II TCP transcription members AtTCP2, AtTCP4 and AtTCP10, the class I TCP transcription factor AtTCP20 induce the expression of *AtLOX2* by binding to its promoter, and therefore decrease the level of jasmonic acid (JA) to inhibit leaf senescence (Schommer et al., 2008; Danisman et al., 2012). Identifying more TCPs will be beneficial in unraveling the precise regulatory mechanisms by which they control plant growth and development.

As one of the most economically important crops, cotton provides the main natural fibers for the textile industry. Cotton fibers are highly elongated ovule epidermal cells that undergo four continuous but overlapping stages: initiation, elongation, secondary cell wall (SCW) biosynthesis and dehydration/maturation (Basra and Malik, 1984; Haigler et al., 2012). It has been shown that the regulation of cotton fiber development depends on multiple plant hormone signaling processes. Auxins, gibberellins and brassinosteroids are required for fiber initiation and elongation both *in vivo* and in *in vitro* ovule culture (Beasley and Ting, 1973; Luo et al., 2007; Xiao et al., 2010; Zhang et al., 2011). Ethylene plays a major role in promoting cotton fiber elongation (Shi et al., 2006). Previous reports also showed that JA suppressed cotton fiber initiation and elongation with sustained high concentrations, while promoted fiber elongation with an appropriate concentration (Hao et al., 2012; Tan et al., 2012). However, the regulatory mechanism upstream of phytohormone signaling in cotton fibers is still not well understood. Our previous research had revealed that GbTCP, homologous to AtTCP15, played positive roles in cotton fiber and *Arabidopsis* root hair elongation by stimulating JA biosynthesis and response (Hao et al., 2012). GhTCP14, which is the ortholog of AtTCP14 in *Arabidopsis*, promoted the differentiation and elongation of trichome and root hair cells in *Arabidopsis* by directly binding to the promoters of auxin-related genes (Wang et al., 2013). These results imply that members of the cotton TCP transcription factor family may be involved in the regulation of fiber development by affecting the synthesis of or response to phytohormones.

Because of their possible roles in fiber development, members of the TCP transcription factor family have been identified and analyzed in different cotton species through an extensive genome-wide survey. A total of 38 and 36 non-redundant TCP genes were identified in diploid *Gossypium raimondii* and *G. arboreum*, respectively (Ma et al., 2014, 2016). A total of 74 and 75 TCP transcription factor members were identified in two allotetraploid cottons, namely *G. hirsutum* and *G. barbadense*, respectively. Quantitative reverse transcription polymerase chain reaction (qRT-PCR) results indicated that many *GhTCPs* and *GbTCPs* were preferentially or specifically expressed in fibers at various developmental stages of cotton. GhTCP14a and GhTCP22 can interact with several transcription factors, which are involved in fiber development (Li et al., 2017; Zheng et al., 2018). *GhTCP4*, a miR319-targeted TCP gene, inhibited fiber cell elongation and promoted cell-wall thickening in cotton (Cao et al., 2020). Heterologous overexpression of the class II TCP genes *GbTCP4* and *GbTCP5* increased root hair length, root hair and trichome density, and the lignin content in transgenic *Arabidopsis* (Wang et al., 2019, 2020). The genome-wide analysis of the TCP transcription factor gene family will lay a solid foundation for future studies into the functional characterization of TCP proteins in cotton fiber development.

In the present study, we performed the genome-wide identification and expression analysis of the TCP family genes in *G. raimondii* slightly different from the previous report (Ma et al., 2014). A class I TCP transcription factor (designated GrTCP11) was cloned based on the phylogenetic and expression analysis, which was preferentially expressed in the stages of cotton fiber initiation and SCW synthesis. An ectopic expression strategy was used to investigate the effect of *GrTCP11* on *Arabidopsis* root hair development, as *Arabidopsis* has been employed successfully as a model system for functional characterization of several cotton fiber-specific genes (Wang et al., 2004; Hao et al., 2012). Overexpression of *GrTCP11* in *Arabidopsis* down-regulated expression levels of JA-related genes, which led to lower JA concentration and shorter root hair cells. In addition, we found that GrTCP11 also affects the bolting and flowering time of *Arabidopsis*. Our findings provide important insights into the role and mechanism of GrTCP11 in cotton fiber and root hair development.

## Materials and Methods

### Plant Materials and Growth Conditions

Cotton plants (*G. raimondii* and *G. hirsutum* cv. TM-1) were cultivated in the field under standard agronomic conditions at Hangzhou Normal University, in Hangzhou, China. Roots, stems and leaves were collected from 15-day-old seedlings. Petals, anthers and stigmas were harvested from flowers at 0 days post-anthesis (DPA). Fibers were detached gently from the ovules at 10 to 25 DPA. The collected materials were immediately frozen in liquid nitrogen and stored at –80°C prior to use.

The *Arabidopsis thaliana* wild-type (WT) was a Columbia-0 accession and the JA-deficient mutant *dde2-2* was derived from Columbia-0. Seeds were first vernalized at 4°C for at least 2 days and then germinated in an illuminated growth chamber (22°C, 16-/8-h light/dark cycle regime) in soil or on agar plates (after surface sterilization of the seeds) containing half-strength Murashige and Skoog (MS) salts.

### Identification of TCP Genes From *Gossypium raimondii*

The reference genome of diploid cotton *G. raimondii* was downloaded from the Joint Genome Institute (JGI) Phytozome.^1^ The TCP protein sequences of *Arabidopsis* were downloaded from NCBI^2^ and used as reference sequences for building a Hidden Markov Model (HMM) to identify the *G. raimondii* TCP proteins using HMMER 3.1.^3^ All the putative TCPs were further subjected to SMART^4^ and InterPro^5^ analyses to identify their conserved domains as previously described (Long et al., 2020). The Compute pI/Mw tool^6^ was used to calculate the molecular weight (Mw) and theoretical isoelectric point (pI). Multiple sequence alignments were performed using Clustal X (version 1.83) (Thompson et al., 1997). The phylogenetic tree was generated from the aligned sequences, using the neighbor-joining method in MEGA6, with 1000 bootstrap replicates (Tamura et al., 2011).

### Heat-Map Analysis

Transcriptome data of *G. hirsutum* cv. TM-1 were downloaded from SRA databases (Accession code: PRJNA248163) (Zhang et al., 2015). Various tissues (root, stem, leaf, petal, pistil, stamen, ovule, and fiber) and abiotic stress (cold, hot, drought and salt stress for 1, 3, 6, and 12 h) transcriptome datasets were employed. The heat map was generated according to the method described previously (Gao et al., 2018). The reads per kilobase of transcript per million mapped reads values representing the expression levels of TCPs were collected from the “TM-1” cultivar transcriptome data downloaded from NCBI. The gene expression data were analyzed using the Genesis 1.8.1 program to generate heat maps. The cluster analysis, which was developed using the K-means method on the expression profiles of all 37 TCP genes, was also performed using the Genesis program.

### RNA Extraction and Quantitative Reverse Transcription Polymerase Chain Reaction Analysis

Total RNA was extracted from 0.1 g fresh weight samples with the OmniPlant RNA Kit (DNase I) CW2598 (CWBIO, Beijing, China) according to the manufacturer’s instructions. HiFiScript gDNA Removal cDNA Synthesis Kit CW2582 (CWBIO, Beijing, China) was used to achieve first-strand cDNA synthesis from approximately 1 μg of total RNA. qRT-PCR was performed using the iQ SYBR Green Supermix (Bio-Rad, Hercules, CA, United States) and run on the ABI Prism 7000 system (Applied Biosystems, Foster City, CA, United States). *GhUB7* and *AtACT2* were used as the reference housekeeping genes in cotton and *Arabidopsis*, respectively. The relative expression level of the target genes was normalized against the reference housekeeping genes (Long et al., 2019). The error bars represent the standard deviation of three biological replicates. The primers used in the qRT-PCR are listed in Supplementary Table 1.

### Vector Construction and Plant Transformation

The open reading frame of *GrTCP11* was amplified from *G. raimondii* fiber cDNA with primers GW-GrTCP11F and GW-GrTCP11R (Supplementary Table 2), and inserted into pB7WG2D,1 with the CaMV 35S promoter to generate the overexpression vector *via* the Gateway BP and LR reactions. The construct was electroporated into the GV3101 strain of *Agrobacterium tumefaciens* and used for *Arabidopsis* transformation (Clough and Bent, 1998).

### Observation and Measurements of Root Hair Length

For root hair length measurements, seedlings from surface-sterilized seeds were grown on plates held upright on half-strength MS medium, as previously reported (Hao et al., 2012). Root hairs on 7-day-old seedlings were photographed using an inverted microscope (Nikon Eclipse Ti, Tokyo, Japan), and measured with ImageJ software^7^. At least ten roots were measured for each independent experiment. Data were analyzed by Student’s *t*-test.

### Extraction and Quantitative Analysis of Jasmonic Acid

One-month-old seedlings (500 mg fresh weight) were extracted with 80% cold methanol (v/v) by shaking overnight at 4°C. Each sample was extracted twice to ensure adequate extraction. The organic phase was evaporated to dryness with N_2_ and the residue dissolved in 0.4 mL methanol. The samples were stored at –20°C before being assayed. JA was quantified using an Applied Biosystems 4000Q-TRAR high-performance liquid chromatography–tandem mass spectrometry (HPLC–MS/MS) system with JA (Sigma, St. Louis, MO, United States) as the external standard. Three biological replicates were performed.

### Root Growth Inhibition Assays

Surface-sterilized *Arabidopsis* seeds of the WT, 4-1 and *dde2-2* were grown on plates held upright on half-strength MS medium plates with or without 50 μM methyl jasmonate (MeJA) at 22°C under a 16-h light/8-h dark photoperiod. The effect of MeJA on root growth inhibition was scored after 12 days of growth. Root lengths were measured using a ruler. At least 20 roots were measured for each independent experiment. Data were analyzed by Student’s *t*-test.

## Results

### Identification and Expression Profiles of TCP Genes in *Gossypium raimondii*

The TCP transcription factor family is a group of plant-specific transcription factors that have versatile functions in diverse aspects of plant development, including cotton fiber initiation and elongation (Hao et al., 2012; Wang et al., 2013). Based on an extensive genome-wide survey, we isolated a total of 37 putative non-redundant TCP genes in diploid *G. raimondii* (Table 1 and Supplementary Table 3), different from previous report of 38 non-redundant TCP genes identified from diploid *G. raimondii* (Ma et al., 2014). The deduced protein sequences alignment showed that the counterpart of GrTCP16 was reduced in our identification. Compared with Cotton_D_gene_10033515, the counterpart of GrTCP16 had the identical amino acid sequences, except for a truncation of 136 amino acids at the C-terminus. So, the counterpart of GrTCP16 was discarded as a redundant sequence of Cotton_D_gene_10033515 in our identification. The identities of other TCP proteins exhibit a good correspondence, excluding slight differences between the six proteins (GrTCP1, GrTCP6, GrTCP18a, GrTCP20c, GrTCP22, and GrTCP23) and their counterparts.

**TABLE 1:** TCP gene family in *G. raimondii*.

Gene ID	Length (aa)	MW (Da)	PI
Cotton_D_gene_10000285	300	31988.46	8.65
Cotton_D_gene_1000050	309	34162.31	8.58
Cotton_D_gene_10002307	266	30361.07	7.78
Cotton_D_gene_10002797	338	35440.37	9.08
Cotton_D_gene_10003061	401	43857.08	6.23
Cotton_D_gene_10003308	444	48127.54	6.46
Cotton_D_gene_10003518	488	51182.04	7.37
Cotton_D_gene_10004819	549	57891.27	6.72
Cotton_D_gene_10006406	270	29631.76	9.20
Cotton_D_gene_10006656	501	55904.78	6.67
Cotton_D_gene_10006721	401	43363.73	6.70
Cotton_D_gene_10008391	300	31763.48	8.67
Cotton_D_gene_10008880	344	37542.57	8.85
Cotton_D_gene_10010801	275	29099.25	7.99
Cotton_D_gene_10012543	337	36419.80	6.62
Cotton_D_gene_10016293	418	44326.77	6.90
Cotton_D_gene_10016960	388	41371.47	8.77
Cotton_D_gene_10017148	345	36349.68	8.32
Cotton_D_gene_10017874	353	40581.42	8.65
Cotton_D_gene_10022676	409	43090.37	8.48
Cotton_D_gene_10025316	196	21060.66	8.56
Cotton_D_gene_10027048	285	32029.85	7.23
Cotton_D_gene_10028625	298	31463.08	9.38
Cotton_D_gene_10029652	257	26592.78	9.51
Cotton_D_gene_10029958	327	36256.95	5.80
Cotton_D_gene_10030066	256	26423.56	9.66
Cotton_D_gene_10030346	398	43606.30	9.11
Cotton_D_gene_10030663	361	40855.56	8.20
Cotton_D_gene_10031549	463	50079.77	6.98
Cotton_D_gene_10033147	395	42222.13	8.91
Cotton_D_gene_10033515	352	38234.66	9.37
Cotton_D_gene_10033516	365	39735.14	9.26
Cotton_D_gene_10033598	410	45001.46	7.88
Cotton_D_gene_10034674	243	25363.55	9.99
Cotton_D_gene_10034792	298	31748.51	9.72
Cotton_D_gene_10035275	435	48307.96	6.07
Cotton_D_gene_10039906	409	44213.66	6.78

**FIGURE 1:**
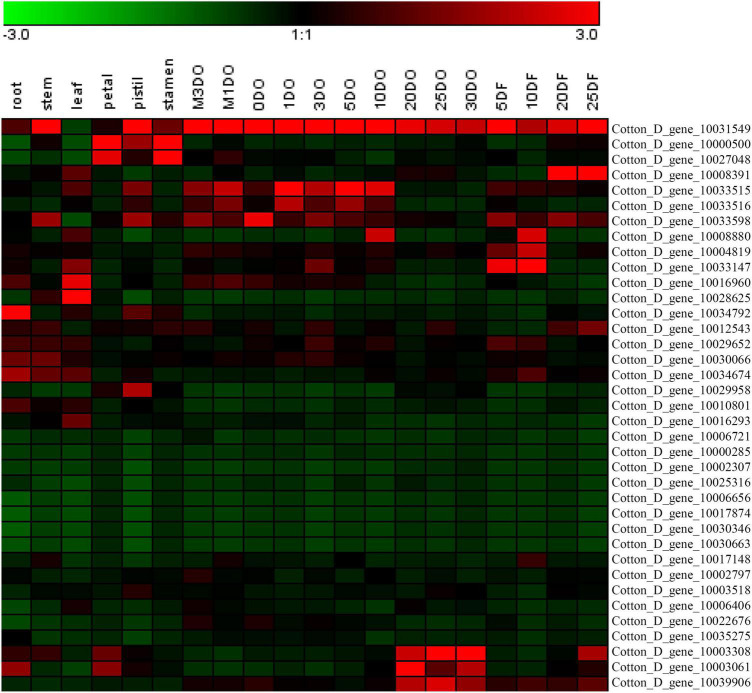
Heat map representation for expression patterns of *Gossypium raimondii* TCP genes in 20 representative tissues. The heat map, generated with Genesis, shows the hierarchical clustering of GrTCPs in root, stem, leaf, petal, pistil, stamen, ovule and fiber tissues. M3DO, M1DO, 0DO, 1DO, and 3DO: ovules attached with fibers at -3, -1, 0, 1, and 3 days post-anthesis; 5DO, 10DO, 20DO, 25DO, and 30DO: ovules without fibers at 5, 10, 20, 25, and 30 days post-anthesis; 5DF, 10DF, 20DF, and 25DF: fibers at 5, 10, 20, and 25 days post-anthesis. The reads per kilobase of transcript per million mapped reads values were log10 transformed and indicated the expression level of *GrTCPs* genes, while the gradient color (red/black/green) reflects the expression levels (high to low).

**FIGURE 2:**
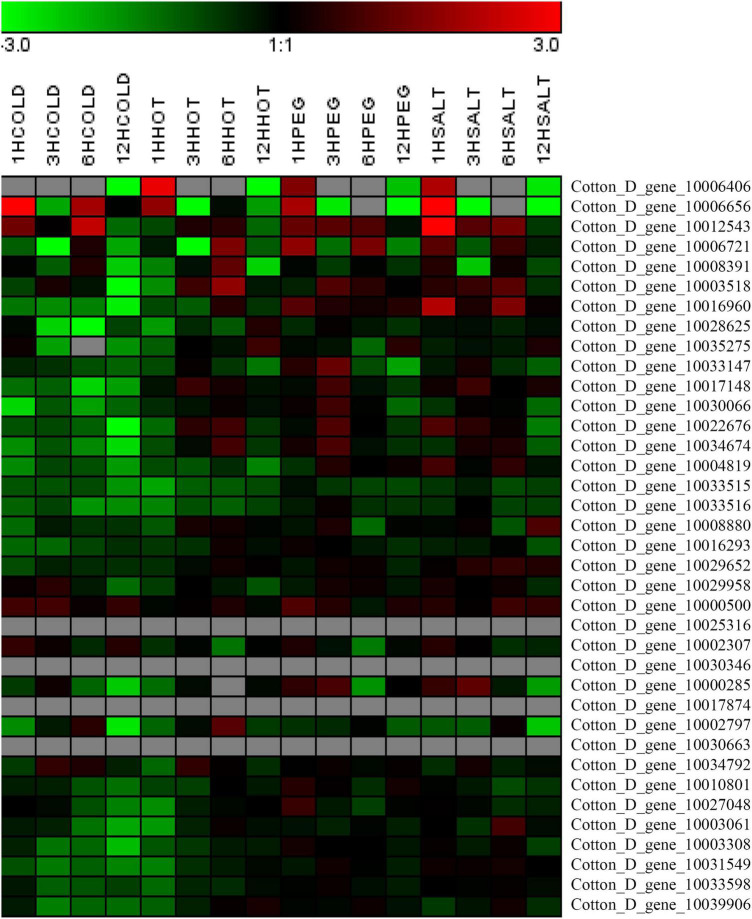
Heat map representation for expression patterns of *Gossypium raimondii* TCP genes in cotton leaves under abiotic stress. The heat map, generated with Genesis, shows the hierarchical clustering of GrTCPs in response to cold (4°C), hot (37°C), drought (20% PEG) and salt (200 mM NaCl) stress, respectively. 1HCOLD, 3HCOLD, 6HCOLD, and 12HCOLD: cold treatment for 1, 3, 6 and 12 h; 1HHOT, 3HHOT, 6HHOT, and 12HHOT: hot treatment for 1, 3, 6, and 12 h; 1HPEG, 3HPEG, 6HPEG, and 12HPEG: drought treatment for 1, 3, 6, and 12 h; 1HSALT, 3HSALT, 6HSALT, and 12HSALT: drought treatment for 1, 3, 6, and 12 h. The reads per kilobase of transcript per million mapped reads values were log10 transformed and indicated the expression level of *GrTCPs* genes, while the gradient color (red/black/green) reflects the expression levels (high to low).

The expression levels of the 37 putative *TCP* genes in different tissues and in response to abiotic stress were analyzed using previously published transcriptome data to understand the possible roles of TCPs (Zhang et al., 2015). The reads per kilobase of transcript per million mapped reads values are listed in Supplementary Tables 4, 5. These values were used to create a heat-map of *TCP* genes’ expression. As indicated in Figure 1, substantial diversity is manifested in the tissue expression profile of TCP members. A few genes (with Gene ID of Cotton_D_gene_10006656, Cotton_D_gene_10017874, Cotton_D_gene_10030346, and Cotton_D_gene_10030663) were expressed at very low level across all 20 tissues, which suggested that they might be primarily expressed under particular conditions. More than half of the remaining *TCP* genes were predominantly expressed in different stages of cotton fiber development, which suggested that these genes might play important roles in fiber development. For example, the genes with Gene ID of Cotton_D_gene_10006406, Cotton_D_gene_10033147 or Cotton_D_gene_10008391 were preferentially expressed during the stages of fiber initiation, elongation or SCW synthesis, respectively. Two genes (with Gene ID of Cotton_D_gene_10000500 and Cotton_D_gene_10027048) had high expression in floral organs but low expression in all other tissues, which indicated they might specifically regulate the reproductive development of cotton. Additionally, the gene with Gene ID of Cotton_D_gene_10031549 was constitutively expressed in every tissue tested at very high level, which implied its possible roles at multiple development stages (Figure 1). However, most TCP members are not very sensitive to abiotic stresses, including cold, hot, drought and salt. For example, four genes (with Gene ID of Cotton_D_gene_10025316, Cotton_D_gene_10030346, Cotton_D_gene_10017874, and Cotton_D_gene_10030663) were not detected in any treatment, which suggested that these genes might not be involved in the stress responses (Figure 2).

### GrTCP11 Was Preferentially Expressed in Anthers and During Stages of Fiber Initiation and Secondary Cell Wall Synthesis

**FIGURE 3:**
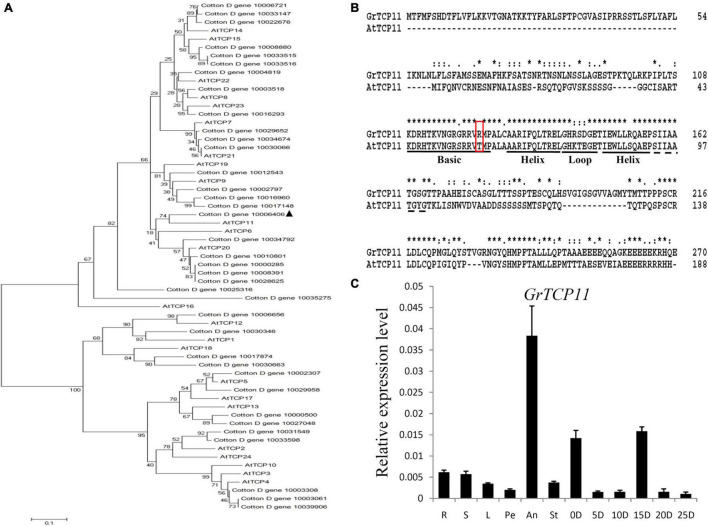
Phylogenetic analysis, sequence alignment and expression analysis of *GrTCP11*. **(A)** Phylogenetic relationships of TCP transcription factors from *Gossypium raimondii* and *Arabidopsis*. The unrooted phylogenetic tree was constructed using MEGA 6.0 with the neighbor-joining method and the bootstrap test involved 1000 iterations. GrTCP11 is marked with a solid triangle. **(B)** Alignment of the amino acid sequences of GrTCP11 and AtTCP15. The horizontal lines indicate the conserved teosinte branched1/cycloidea/proliferating cell factor1 (TCP) domain. The red box indicates the amino acid residue at position 15 of the TCP protein domain. **(C)** Relative expression level of *GrTCP11* in various tissues, including root (R), stem (S), leaf (L), petal (Pe), anther (An), stigma (St) and fibers (0D and 5D: ovules with fibers at 0 and 5 days post-anthesis; 10D, 15D, 20D, and 25 D: fibers at 10, 15, 20, and 25 days post-anthesis) from *Gossypium hirsutum* cv.TM-1; expression of *GrTCP11* was calculated relative to *GhUB7* expression. Error bar on each mean value represents the standard deviation of three biological replicates.

Due to the expression profiles analysis, *GrTCP11* (Gene ID: Cotton_D_gene_10006406) was cloned for further study as one of the fiber-specific expressed genes. Phylogenetic analysis was used to investigate the evolutionary relationship between the TCP proteins from *G. raimondii* and *Arabidopsis*. GrTCP11 was found to belong to class I TCP proteins as a unique homolog of AtTCP11 (Figure 3A). The amino acid sequence alignment showed that the identity of GrTCP11 with AtTCP11 full-length amino acids was 38%, and their TCP domains were 86% identical. Like most class I TCP proteins, the position 15 of the TCP domain in GrTCP11 is arginine, which is different from the threonine in AtTCP11 (Figure 3B). The coding sequence of *GrTCP11* is 813 bp in length and encodes a putative polypeptide of 270 amino-acid residues with a calculated molecular weight of 29.6 kDa and an isoelectric point of 9.20. Based on the alignment between the coding sequence and the genomic DNA sequence, *GrTCP11* was found to consist of two exons and a 366-bp intron. qRT-PCR analysis showed that the transcript level of *GrTCP11* was the highest in anthers, while its transcript level was lower in roots, stems, leaves, stigmas and petals. qRT-PCR also showed that *GrTCP11* was expressed in cotton fibers at different developmental stages. It had the highest transcript level at 0 DPA and 15 DPA, and could not be detected at other stages of fiber development (Figure 3C).

### Overexpression of *GrTCP11* in *Arabidopsis* Inhibited Root Hair Elongation and Delayed Flowering Timing

**FIGURE 4:**
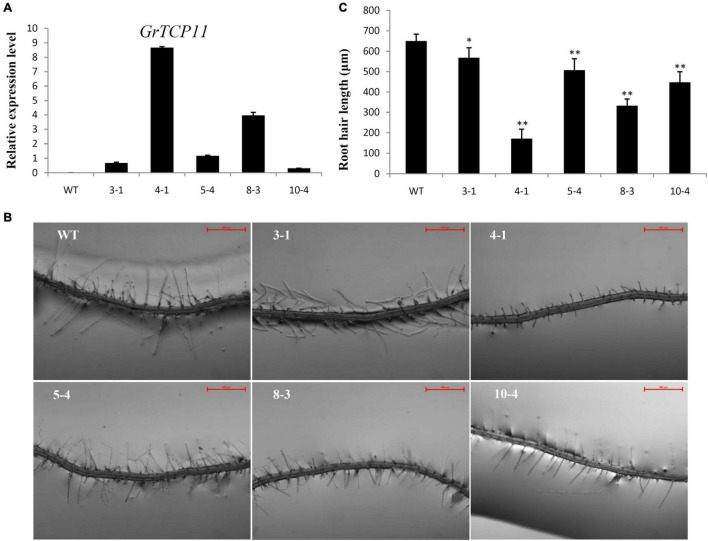
Expression level and morphological alterations of the root hairs in *Arabidopsis* transgenic plants overexpressing *GrTCP11*. **(A)** Relative expression level of *GrTCP11* in transgenic lines (3-1, 4-1, 5-4, 8-3, and 10-4) and wild-type plants; expression of *GrTCP11* was calculated relative to reference *AtACT2* expression; error bars represent the standard deviation of three biological replicates. **(B)** Root hairs in the mature area of taproots of 7-day-old transgenic lines and wild-type plants; scale bars = 500 μm. **(C)** Length of the root hairs of 7-day-old seedlings within the mature zone; data are mean of 100 root hairs; *P < 0.05; **P < 0.01 relative to the wild-type, using Student’s *t* test.

**FIGURE 5:**
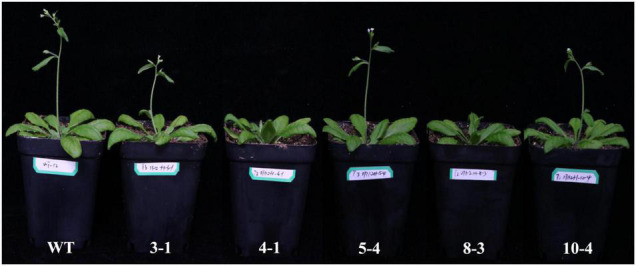
The flowering time in 1-month-old wild-type and *GrTCP11*-overexpressed transgenic lines (3-1, 4-1, 5-4, 8-3, and 10-4).

To verify the function of *GrTCP11*, we generated ectopic *GrTCP11*-overexpressed transgenic *Arabidopsis* plants, to bypass the difficulty and delay associated with conventional cotton transformation. Several independent transformants were isolated and the expression levels were measured using qRT-PCR. Five independent T_3_ transgenic homozygous lines (3-1, 4-1, 5-4, 8-3, and 10-4) with different expression levels were further analyzed (Figure 4A). Overexpression of *GrTCP11* in *Arabidopsis* inhibited root hair elongation. The mean length of the root hairs was significantly shorter in transgenic overexpressing lines 4-1 (171.2 μm), 8-3 (332.6 μm), 10-4 (448.3 μm), 5-4 (507.2 μm) and 3-1 (568.1 μm) than the root hairs of WT plants (650.3 μm) (Figures 4B,C). Overexpression of *GrTCP11* in *Arabidopsis* also influenced the timing of flowering. The flowering time of the WT plants was earlier than in the transgenic lines (Figure 5). But there was no difference in their maturity time (Supplementary Figure 1).

### Up-Regulation of *GrTCP11* Affected Transcription of Genes Involved in Jasmonic Acid Biosynthesis and Response

**FIGURE 6:**
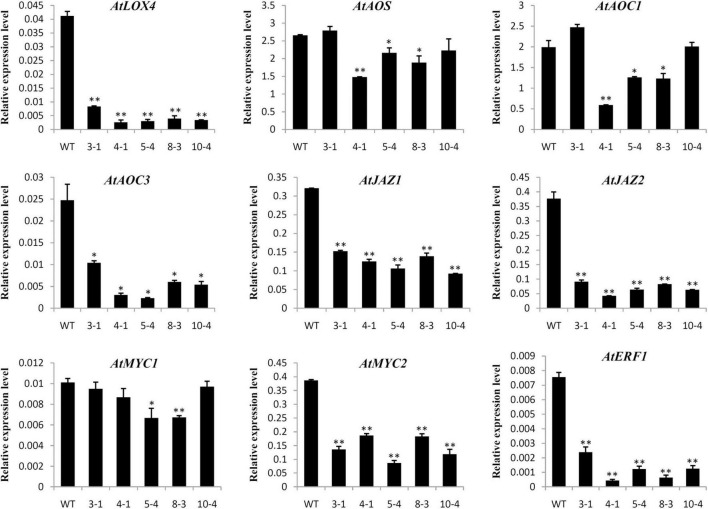
Relative expression level of the genes related to JA biosynthesis and response in wild-type and *GrTCP11*-overexpressed transgenic lines (3-1, 4-1, 5-4, 8-3, and 10-4). The accession numbers of the genes are as follows: AtLOX4, AT1G72520; AtAOS, AT5G42650; AtAOC1, AT3G25760; AtAOC3, AT3G25780; AtJAZ1, AT1G19180; AtJAZ2, AT1G74950; AtMYC1, AT4G00480; AtMYC2, AT1G32640; AtERF1, AT3G23240. Gene expression values are relative to reference *AtACT2* expression; error bars represent the standard deviation of three biological replicates. Asterisks indicate a significant difference (*P < 0.05; **P < 0.01) relative to the corresponding wild-type, using Student’s *t* test.

Previous studies indicated that TCP transcription factors could regulate the development of cotton fibers and *Arabidopsis* root hairs through mediating phytohormone biosynthesis and signal transduction (Hao et al., 2012; Wang et al., 2013). Therefore, the expression levels of genes involved in phytohormone biosynthesis and response were determined in transgenic overexpressing lines and WT plants by qRT-PCR. As shown in Figure 6, many genes associated with JA biosynthesis (*AtLOX4* and *AtAOC3*) and response (*AtMYC2*, *AtJAZ1*, *AtJAZ2*, and *AtERF1*) were significantly down-regulated in *GrTCP11*-overexpressed transgenic lines, compared with the WT plants. However, there was no significant difference in the expression levels of genes associated with other phytohormones, such as *AtACO2*, *AtETR1*, *AtCTR1*, *AtTAA1*, *AtPIN1*, *AtIAA3*, *AtITP1*, *AtAHK3*, and *AtARR1* (Supplementary Figure 2).

### Induced Expression of *GrTCP11* Reduced Jasmonic Acid Concentrations in *Arabidopsis*

**FIGURE 7:**
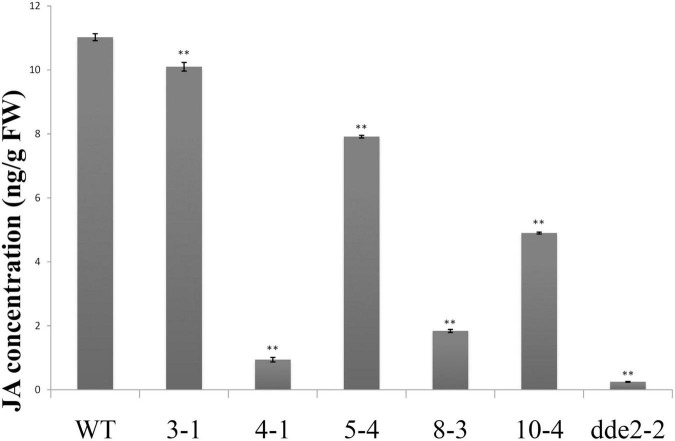
JA concentration in wild-type, *GrTCP11*-overexpressed lines (3-1, 4-1, 5-4, 8-3, and 10-4) and the JA-insensitive mutant *dde2-2*. Three independent experiments were performed; error bars represent the standard deviation. Significant differences between the wild-type and other lines were calculated by Student’s *t*-test analysis (**P < 0.01).

Due to changes in the expression of JA biosynthesis and signaling-related genes, it was hypothesized that GrTCP11 might affect root hair development by regulating JA concentrations. To test this hypothesis, we extracted and determined the concentration of JA in transgenic lines overexpressing *GrTCP11*, *dde2-2* and WT plants, using HPLC–MS/MS. The results showed that the concentrations of JA were significantly lower in the overexpressed lines, compared with the WT controls. In particular, the JA concentration in line 4-1, which had the lowest expression level of JA-related genes, was similar to that in the mutant *dde2-2* (Figure 7).

### Induced Expression of *GrTCP11* Decreased the Sensitivity of Root Growth to Methyl Jasmonate

**FIGURE 8:**
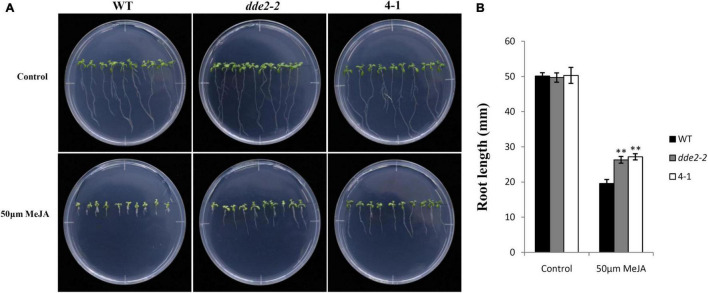
Sensitivity analysis of wild-type, transgenic line 4-1 and JA-insensitive mutant *dde2-2* in response to methyl jasmonate (MeJA). **(A)** 12-day-old seedlings were grown on agar plates supplemented with (50 μM MeJA) or without (Control) MeJA. **(B)** Measurement of the root length in wild-type, 4-1 and *dde2-2* grown for 12 days on agar plates held upright and containing or not containing 50 μM MeJA. Data are mean of at least 20 roots; error bars represent the standard deviation of 20 biological replicates; **P < 0.01 relative to the wild-type, using Student’s *t* test.

To further test the effect of GrTCP11 on JA biosynthesis in *Arabidopsis*, 12-day-old seedlings of WT, 35S:*GrTCP11* transgenic line 4-1 and *dde2-2* were treated with 50 μM MeJA. As shown in Figures 8A,B, before MeJA treatment, 4-1 and *dde2-2* were similar to WT plants in terms of root length. In the presence of 50 μM MeJA, the root growth of 4-1 was as insensitive to MeJA as was *dde2-2*. However, WT plants were hypersensitive to MeJA, with significantly shorter roots.

## Discussion

### GrTCP11 Might Regulate Cotton Fiber Development by Directly Down-Regulating Jasmonic Acid Biosynthesis and Response

Several TCPs have been shown to function in the control of trichome and root hair development (Hao et al., 2012; Vadde et al., 2018; Wang et al., 2019, 2020). In the present study, a cotton class I TCP transcription factor GrTCP11 was characterized, which was homologous to AtTCP11. Heat-map and qRT-PCR analysis showed that *GrTCP11* was preferentially expressed during the stages of fiber initiation and SCW synthesis, rather than in the fiber elongation stage, which indicated that GrTCP11 had a spatio-temporal regulatory effect on cotton fiber development. Ectopic overexpression of *GrTCP11* in *Arabidopsis* led to shorter root hair length. Previous studies have shown that there may be similar regulatory mechanism operating between the elongation of cotton fibers and *Arabidopsis* root hairs (Hao et al., 2012). We speculated that GrTCP11 might also inhibit cotton fiber elongation.

Plant hormones play vital roles in fiber and root hair development, as previously described. The expression levels of genes associated with JA biosynthesis and response (*AtLOX4*, *AtAOC3*, *AtJAZ1*, *AtJAZ2*, and *AtMYC2*) were significantly down-regulated in the transgenic lines compared with the WT controls, in association with a decrease in JA concentration. Changes in JA concentration were further validated with respect to JA sensitivity and flowering time. It has been reported that MeJA inhibits root growth in *Arabidopsis*, whereas the sensitivity to MeJA can be alleviated in some JA-deficient mutants (Staswick et al., 1992; Lorenzo et al., 2004). Compared to the wild type, the transgenic line 4-1 exhibited reduced sensitivity of root growth to 50 μM MeJA, similar to that of the JA-deficient mutant *dde2-2*. Jasmonic acid has been implicated in regulating flowering time (Pak et al., 2009). The flowering times of transgenic lines 4-1 and 8-3 were later than the other lines, which might be associated with the decreased JA concentration in these transgenic lines. AtERF1, a common downstream responsive element of the jasmonate and ethylene pathways, was also significantly suppressed by GrTCP11 (Lorenzo et al., 2003). However, no significant difference was observed in the expression levels between the transgenic lines and WT plants of other hormone-related genes, involved in the biosynthesis and response of ethylene, auxin or cytokinin. The above results revealed that GrTCP11 might inhibit fiber and root hair elongation by directly suppressing JA biosynthesis and response. JA is a crucial factor which suppresses cotton fiber initiation, with an appropriate concentration promoting fiber elongation as described above. Therefore, the expression pattern of *GrTCP11* is associated with fiber initiation and elongation. Overall, spatio-temporal specific *GrTCP11* may be an important regulator for cotton fiber development through negative regulation of JA biosynthesis and response.

### The Possible Molecular Mechanism of TCP Transcription Factors in Developing Cotton Fibers

Cotton is one of the most important economic crops in the world, as its main product, cotton fibers, are the main raw material for the natural textile industry. Previous studies had revealed that transcription factors were widely involved in the regulation of cotton fiber development (Samuel Yang et al., 2006; Hande et al., 2017). Most of the plant-specific R2R3-MYBs were shown to regulate various developmental stages in cotton fiber, such as GhMYB109, GhMYB25 and GhMYB25-like (Pu et al., 2008; Machado et al., 2009; Walford et al., 2011). A R3-MYB transcription factor GhCPC delayed fiber initiation and inhibited early elongation by a potential CPC-MYC1-TTG1/4 complex. GhMYC1 could bind to the E-box *cis*-elements and the promoter of *GhHOX3*, which suggested that *GhHOX3* may be downstream gene of the regulatory complex (Liu et al., 2015). GbTCP and GhTCP14 were shown to play vital roles in fiber initiation and/or elongation through regulating JA- and auxin-related genes, respectively, opening up research into the role of TCP transcription factors on cotton fiber development (Hao et al., 2012; Wang et al., 2013). Genome-wide analysis of the TCP family in a number of cotton varieties showed that they might be involved in different physiological processes of cotton fiber development. It was found that GhTCP22 and GhTCP14a could interact with several transcription factors related to cotton fiber growth and development, such as GhSLR1, GhGL3, GhARF6, GhTTG1, GhMYB23, and GhMYB25. In addition, GhTCP14a could also interact with GhEIN3, GhBZR1, and GhMYB25-Like proteins (Li et al., 2017). GhTCP4 interacted with GhHOX3 to coordinate fiber cell elongation and SCW biosynthesis, two events that are key to cotton fiber traits (Cao et al., 2020). Overexpression of *GbTCP4* increased root hair length, root hair and trichome density, and the lignin content by binding directly to the *AtCPC* and *AtCAD5* promoters in *Arabidopsis* (Wang et al., 2019). GbTCP5 regulated root hair development and SCW formation by binding to the promoters of the *AtGL3*, *AtEGL3*, *AtCPC*, *AtMYB46*, *AtLBD30, AtCesA4*, *AtVND7*, *AtCCOMT1*, and *AtCAD5* genes to upregulate their expression in *Arabidopsis* (Wang et al., 2020). Here, we found that GrTCP11 possibly regulate fiber development by inhibiting JA biosynthesis and response in fiber initiation and SCW synthesis. The results indicated that TCP transcription factors possibly regulated fiber development by interacting with other fiber-related transcription factors, or binding to promoters to activate or inhibit their expressions. Protein complexes or targeted genes may regulate fiber development by affecting the homeostasis of phytohormones.

### TCP Proteins Show Functional Diversity in Different Species

A growing number of TCP transcription factors have been characterized and confirmed to be widely involved in the regulation of plant growth, architecture and development. TCP transcription factors have some evolutionarily conserved roles in a range of plant species, such as regulation of branching, floral symmetry and leaf development (Danisman, 2016). For example, TEOSINTE BRANCHED1 in maize and its orthologs in rice, *Arabidopsis*, pea and poplar have a conserved function of suppressing branching (Doebley et al., 1997; Aguilar-Martinez et al., 2007; Braun et al., 2012; Muhr et al., 2016). On the other hand, there are suggestions that TCP proteins may have some new evolutionary roles in different species. Two closely related *Arabidopsis* TCP transcription factors, TCP14 and TCP15, were shown to be redundant in promoting cell proliferation in internodes and trichomes, and repressing cell proliferation in leaves and flower tissues (Kieffer et al., 2011). They were also necessary for seed germination by gibberellin-dependent activation (Resentini et al., 2015). AtTCP15 could modulate gynoecium development by participating in a feedback loop that helps to adjust the balance between auxin levels and cytokinin responses (Lucero et al., 2015). However, their homologous proteins in cotton had new biological functions. GbTCP, a protein orthologous to AtTCP15 in Sea-island cotton, promoted cotton fiber and *Arabidopsis* root hair elongation, as well as plant branching by directly activating the biosynthesis of and response to JA and then affecting downstream complex signal regulatory networks (Hao et al., 2012). Ectopic expression of *GhTCP14* from upland cotton, which is homologous to *AtTCP14*, promoted the differentiation and elongation of trichomes and root hair cells through alteration of auxin homeostasis (Wang et al., 2013). *AtTCP11* influences the growth of leaves, stems and petioles as well as pollen development in *Arabidopsis* (Viola et al., 2011). In the current work, a homolog of *AtTCP11*, named *GrTCP11*, was characterized in *G. raimondii*. High transcript abundance in anthers implied that *GrTCP11* may have the same conserved function in pollen development as did *AtTCP11*. Our results showed that *GrTCP11* may affect fiber and root hair elongation by directly inhibiting JA biosynthesis and response. The above research indicated that TCPs share some common functions but have also partially diverged, evolving distinct roles in different species through different regulatory mechanisms.

## Data Availability Statement

The datasets presented in this study can be found in online repositories. The names of the repository/repositories and accession number(s) can be found in the article/Supplementary Material.

## Author Contributions

JH and MX designed the experiments. PL, YH, ZC, and JC performed the experiments. JN, YY, and ZJ performed the data analyzes. JH wrote the manuscript. All authors read and approved the final manuscript.

## Conflict of Interest

The authors declare that the research was conducted in the absence of any commercial or financial relationships that could be construed as a potential conflict of interest.

## Publisher’s Note

All claims expressed in this article are solely those of the authors and do not necessarily represent those of their affiliated organizations, or those of the publisher, the editors and the reviewers. Any product that may be evaluated in this article, or claim that may be made by its manufacturer, is not guaranteed or endorsed by the publisher.

## Footnotes

1
https://phytozome.jgi.doe.gov

2
http://www.ncbi.nlm.nih.gov/

3
http://hmmer.janelia.org/

4
http://smart.embl-heidelberg.de/

5
http://www.ebi.ac.uk/interpro/

6
http://web.expasy.org/compute_pi/

7
http://rsbweb.nih.gov/ij/

## Supplementary Material

Supplementary Figure 1 The maturity in 2-month-old wild-type (WT) and *GrTCP11*-overexpressed transgenic lines (3-1, 4-1, 5-4, 8-3 and 10-4).

Supplementary Figure 2 Relative expression level of the genes related to phytohormones biosynthesis and response in wild-type (WT) and *GrTCP11*-overexpressed transgenic lines (3-1, 4-1 and 8-3). Gene expression values are relative to reference *AtACT2* expression; error bars represent the standard deviation of three biological replicates.
