# Supplementary figures and images for: GrTCP11, a Cotton TCP Transcription Factor, Inhibits Root Hair Elongation by Down-Regulating Jasmonic Acid Pathway in Arabidopsis thaliana
Source: Front Plant Sci. 2021 Nov 22;12:769675. doi: 10.3389/fpls.2021.769675 (PMC8646037; doi:10.3389/fpls.2021.769675)

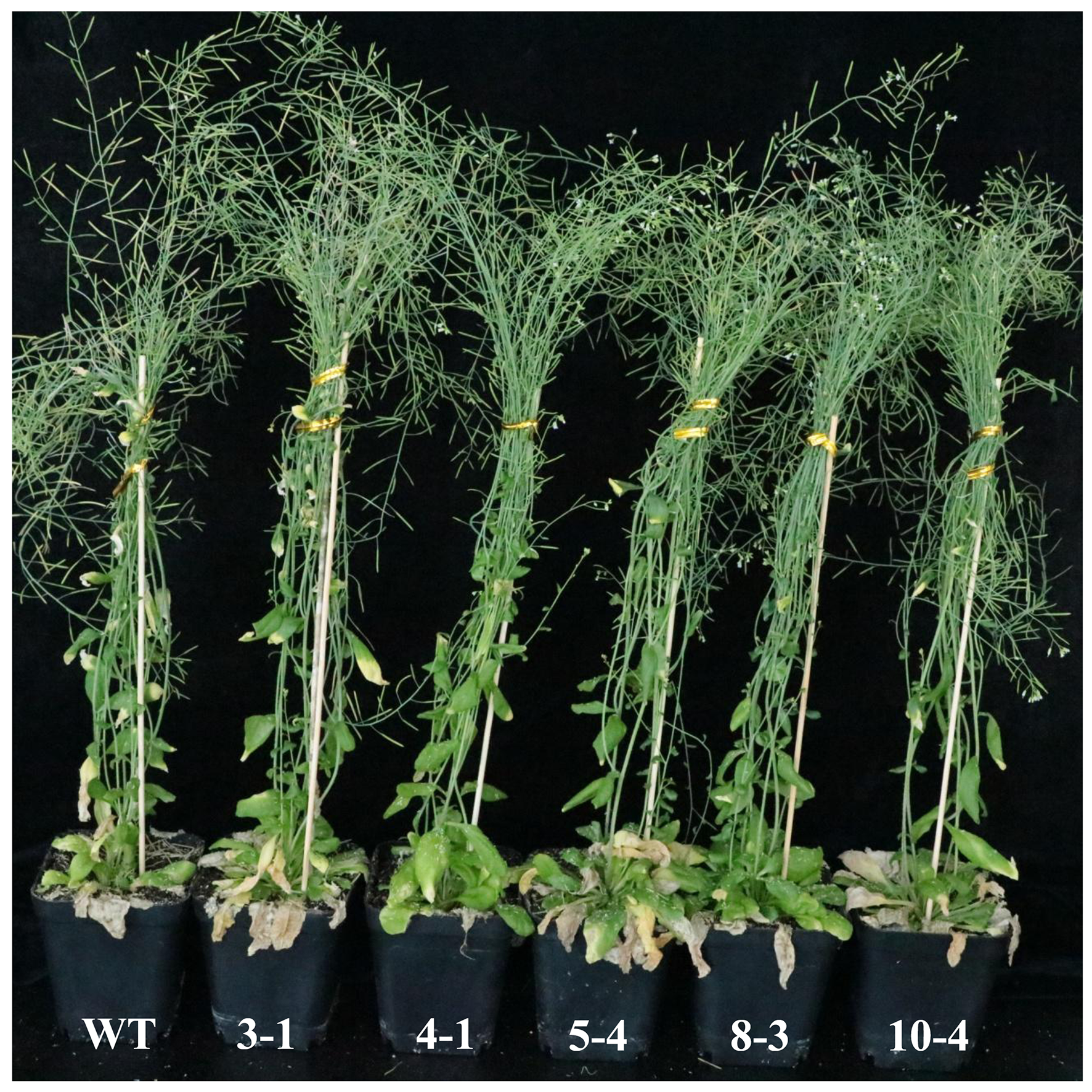

Supplement: Supplementary Figure 1 — The maturity in 2-month-old wild-type (WT) and GrTCP11-overexpressed transgenic lines (3-1, 4-1, 5-4, 8-3 and 10-4). [file Image_1.TIF]

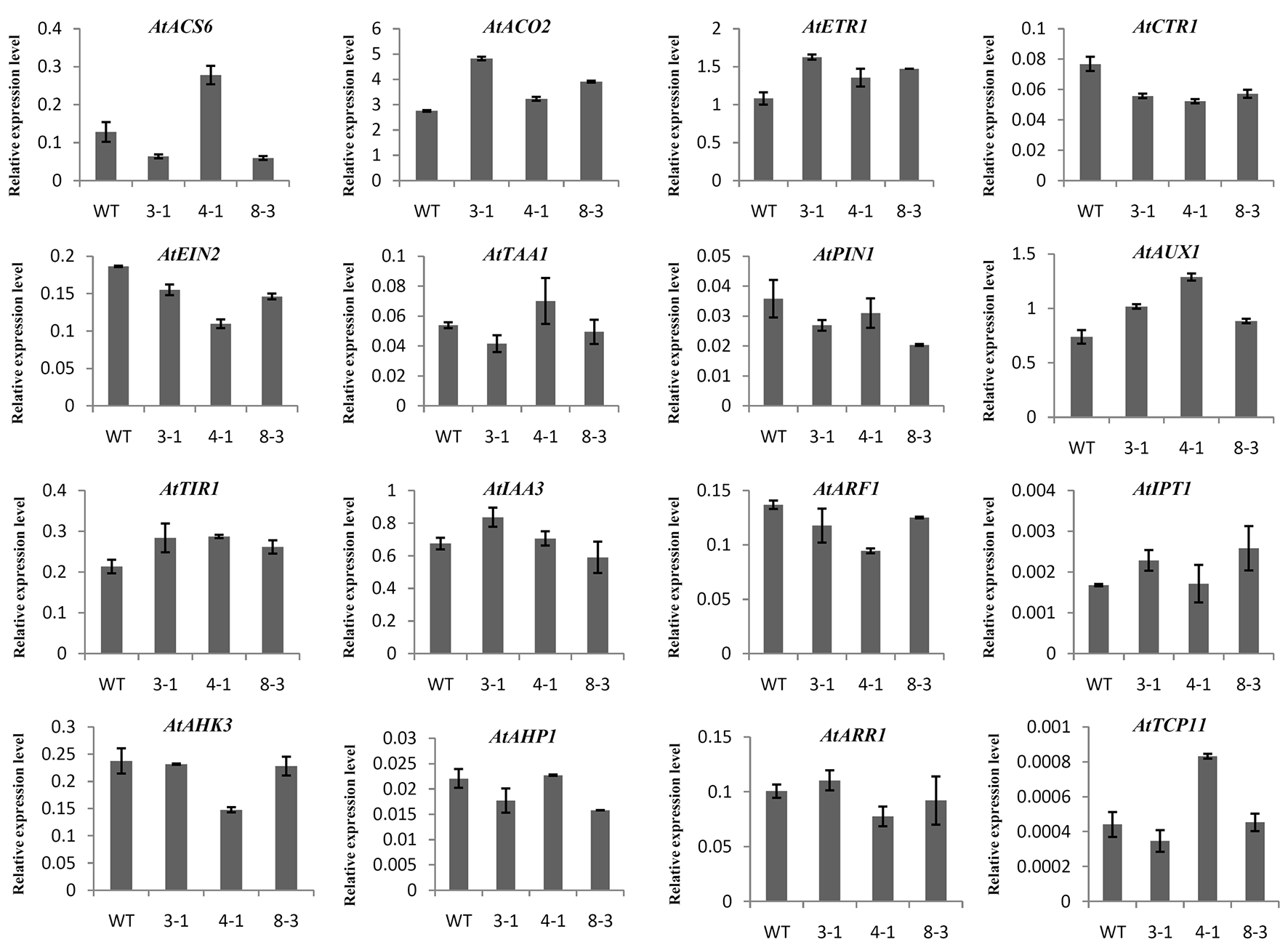

Supplement: Supplementary Figure 2 — Relative expression level of the genes related to phytohormones biosynthesis and response in wild-type (WT) and GrTCP11-overexpressed transgenic lines (3-1, 4-1 and 8-3). Gene expression values are relative to reference AtACT2 expression; error bars represent the standard deviation of three biological replicates. [file Image_2.TIF]
