# Supplementary material for: GrTCP11, a Cotton TCP Transcription Factor, Inhibits Root Hair Elongation by Down-Regulating Jasmonic Acid Pathway in Arabidopsis thaliana
Source: Front Plant Sci. 2021 Nov 22;12:769675. doi: 10.3389/fpls.2021.769675 (PMC8646037; doi:10.3389/fpls.2021.769675)
Supplement: Supplementary file 5 [file Table_3.DOCX]

Supplementary Table S3. Sequences of the GrTCP proteins.

>Cotton_D_gene_10000285

MDSKQSPKKVANFLSLPQQQQQPQNMGENKVGEVQDSQIVIADKEGKKQQLAPKRSSNKDRHTKVEGRGRRIRMPALCAARIFQLTRELGHKSDGETIQWLLQQAEPSIIAATGSGTIPASALAAAGGSVSQQGASLSAGLHQRLEDLGGSSSSIGSGSGRTSWGIEGNLGRPHHVGTGLWSPVSGYGFQSLSGPSTTNLGTESSNYLQKLGFPGFDLPPTNMGQVSLTSILGAANEQQLPGLELGLSQEGHIGGLNPQALSQFYQQMGQARVHQQEQYQHQHQHQHQPPPKDDSQGSGQ

>Cotton_D_gene_10000500

MIPSSEEAGSPIQEGKEDESSNKVRKGASTSSPLLRLKDPRIVRVSRAFGGKDRHSKVFTVRGLRDRRVRLSVPTAIQLYDLQDRLGLNQPSKVVDWLLNEAKHEIEELPPLPIPQGNFSLNPQMPLNASQQPNKQDSTGGFCRAKSKDIVRESSKGSKRDEEASEGHQVQTDYGLQRSNHSPMPALSNNAVSMPMPMPMPYGSYYHFEPSNFPLPHLGSHGFVAPQTEEVHNFNVVPLSSTLSLSSGSQNHFQMLSSGAQNPFANPPQYSITQPDLRSFHLSIAPRLLPHSLNINGSQPGKEDEFPSK

>Cotton_D_gene_10002307

MISSSREKSCFEAKQEGDGGKVSSSRQWQGFRNPRIVRVSRSFGGKDRHSKVCTIRGLRDRRIRLSVPTAIQLYDLQDRLGLNQPSKVIDWLLEATKDDVDKLPPLEFHHSLMPNPCQSSPFTPLMDPNLMFMKDYEGETSMQVEREITDMKGKWIKEQEKLIFPVTNHGSSLPGGFMFNTGTNSMPLNTYNHHWNPSQFTNHGFHHHHQPENYFHGAIANTYPPLPSCPPPSTTPSHFPTFPWYGTNTNQDAAGDQDNSKLQFFT

>Cotton_D_gene_10002797

MAAIHKLEVEEDADPRAVVLSINGGGDAAPSKNPKEEPDIDTTLGAMPTAVHVPSGVPVPPVTAAPAAPKRASTKDRHTKVEGRGRRIRIPATCAARIFQLTRELGHKSDGETIRWLLEHAEPAIIAATGTGTVPAIAMSVNGTLKIPTTSNANPEPGDPSKKKHKRPANSEYVDINDAVSVSSGLAPVVTPQQQQQQQAAVLPQGLVPIWAIPSNTVVPGAFFMVPPMASMAGPSTQPHIFTFPATATPVINISARPISSFVSAMQAATPSQLQSNVAVTSCTVPVSKAAKTTSVMAPSSSSAASATTTTTTQMLRDFSLEIYDKQELQFMTRSSKH

>Cotton_D_gene_10003061

MGENHHQAAATSSRLRIKHVGGEIVEVQGGHIVRSTGRKDRHSKVCTAKGPRDRRVRLSAHTAIQFYDVQDRLGYDRPSKAVDWLIKKAKSAIDELAELPPWNPLDATISTKKPNNQEDLNTSTATNNEFHIENLAAGSGSQCVQQHEMGDNLNNNSGFLPASLVSDEIADTMKSFFPLGASSETPPSSSLQFQDYPPDLLSRTSSHSQDLRLSLQSFPEPILLHHHEHHAAATAQAHHSESVLFSGTTPLAGFDGSNAGWEHHQQQQPVEIGRFQRLFAWNSSGGDAGGGNGGGFIFGTPSQQSLPLTFGQNSQFFSQRGPLQSSNTPLVRAWIDQPIDEHHHHHQIPQNIHQQAALSSMGFTTSGVYSGFRVPSRIQGQEEEHDSIANKLSSASSDSHH

>Cotton_D_gene_10003308

MGDSHHQAATSSRLGIRHSGGEIVEVQGGHIVRSTGRKDRHSKVCTAKGPRDRRVRLSAHTAIQFYDVQDRLGYDRPSKAVDWLIKKAKPAIDELAELPPWNPETLNTTTSIAKTSNQEDQNATTATDNDKPYQFHINHSGNLVENLAAGSGTRRRTATIMGNEVQSLQQQEMGDNPNNNSGFLPPSLVSDEIADTIKSFFPVGASSETPSSSIQFQNYPPDLLSRTSSHSQDLRLSLQSFPEPILLHHHHHHAQAATAQAHHSEPVLFSGSSPLAGFDGSSAGWEHHHQHPAEIGRFQRLFAWNNSGAAADSSGGGGAGGSSGVGGGFIFGTPPPQSLPPAFGQNSQLFSQRGPLQSSNTPLVRAWIDQPISTTDQHQHHHHQQHHHHQIPENIHHHTALSGIGFTTPGVFSGFRVPARIQGEEDEHDSIANKLSSASSDSHH

>Cotton_D_gene_10003518

MELTYSQNNKQTNNNTNLVSFDGTTTTSSAPSSTSNNNNSPFNITSISHLHRRLLHPTTSSTATVITTATPPLSSSSSSSSSSTTKTNPAQPVDASLAIATRSSASLNIDDSTKKNQPNLPPSSNPPAKRSTKDRHTKVDGRGRRIRMPAACAARVFQLTRELGHKSDGETIEWLLQQAEPAIIAATGTGTIPANFSTLNVSLRSSGSTLSAPPSKSAPHSFHGALALASHHHPYEEGFAHSALFGFHHQQQQLLRAGQIAETLPAGGSGSGGGGNSSENYMRKRFREDLFKDDNQQQGETGDGGGSGDGDSGGDGSSIKAFKTGLPQLHKPSNILPATAMWAVAPAPSSTTGSTFWMLPVTTTGPSILTAASGAGSSEPQMWPFGTASGNTLQAPLHFVPRFNLQGDLESQGGRASPLQLGSMLLQQQHQPSQHLGLGLSESNLGMLAALNAYSRGGPNINSDQNNPMEHHQQQGTNSGDENPNNSQ

>Cotton_D_gene_10004819

MELTDLQSNKQTKNNSSSSNNNNNNSAEPHHQSLQQHHLQHQKQSASPQLVVPFDGTRSSGPSSGNPFNMGSISHLHPHHPFHHHLLHPASSTTTTTTTPPLPSSSTSSSSSSSASTTTTNPPQLVDASLAIATRSTSLNIDSKNQTDLPISSTTTPASTTTTTTTANPPVKRSTKDRHTKVDGRGRRIRMPATCAARVFQLTRELGHKSDGETIEWLLQQAEPAIIAATGTGTIPANFSTLNISLRSSGSTLSAPPSKSAPHSFHGALALASHHHHHHPYEEGFAHSALLGFQQQHLLTADQIAEALPGGGGGDGGNLSENYMRKRFREDLFKDDDQQQGESGSGGGDGSPIKAFKSGLTQLSKPQQDAGSSALPRPSNILPGTAMWAVAPAPSSGAGSTFWMLPMSAGAGGPSVAPSAATGAGPSDHPQMWPFGTANQASGNTLQAPLHFLPRFNLPGNVEFQGGRASPLQLGSMLMQQQQQQQQPHHHLGLGMSDSNLGMLAALNAYSRGGSNVNSDQNNPLEHHHQQHQPQGTDSGDDQDPNTSQ

>Cotton_D_gene_10006406

MTFMFSHDTFLVFLKKVTGNATKKTYFARLSFTPCGVASIPRRSSTLSFLYAFLIKNLNLFLSFAMSSEMAFHKFSATSNRTNSNLNSSLAGESTPKTQLRKPIPLTSKDRHTKVNGRGRRVRMPALCAARIFQLTRELGHRSDGETIEWLLRQAEPSIIAATGSGTTPAAHEISCASGLTTTSSPTESCQLHSVGIGSGVVAGMYTMTTPPPSCRLDLCQPMGLQYSTVGRNGYQHMPFTALLLQPTAAAEEEEQQAGKEEEEEKRHQE

>Cotton_D_gene_10006656

MFPSSNSYNPCPSTIHAMPGNSISADQHPADSSLEDHPPPFFNFPASFFDDDDDDGLLMSHLLSQAQQQILGSSSNTAPPDSEINAAPPIKETKKVPPNRKRSSSNGAKQGIPRKRTGKKDRHSKIYTAHGPRDRRMRLSLQIARKFFDLQDMLGFDKASKTIEWLFSKSKAAIKELTENLPALKHRCSEGGKSVSSTSESEVVSAAKECQDNMGDQQGIIASGEAMRSTTTARVTKERKSRKVSFNHIVARESRDKARARARERTREKMRMRDLEKTNKCSDESNPNELEELQSSSPLEIGENSGPSTETNYSLKVVTEKPHRNTADSDSIEHQMDFVSTVEKFLGITRSSSMFNYSNNIADSREENSAENCPVFSGNWGMNNERIHYSYCAMTNIKDSTGITQEQNPSIIFMTDPNEQEQKFSSSFMSNSNTQAENSTTNLIANSYAKMMNHNSDLTNPSNAHEGRNPTSILMSSSHIGLHSDYHENPAVASKFHHFYL

>Cotton_D_gene_10006721

MEGGGSDDHQLHHHRHNHHHRPTFPFQLLEKKEEDNQPCSSSSSPTFPSMALSSSSADQTNTPTSISALQISPDPSKKPPPKRTSTKDRHTKVDGRGRRIRMPALCAARVFQLTRELGHKSDGETIEWLLQQAEPAVIAATGTGTIPANFTSLNISLRSSGPSMSVPSQLRSSGFNSNFSMQQRRSLFPGIGLETTPTFLNFQSNTSSNLNFMFQTKQELRDNDGTSLEISETEEGVLGRKRRPDQDLSSQHQMGSYLLQSSTGEIPAASHGQIPANFWMVTNSNNQVMSADRDPVWTFPSVTNSALYKGPMSSGLHFMNFPTPMALLSGQQLGSSSVSSGGVGDGSSGLSEGHFNMLAGLDPYRQVSGTVVSGSQVSRSHSHHDGAGGGDDRHDTTSHHS

>Cotton_D_gene_10008391

MDPKGAKQPPEEVANLLSLPPQPQQQQPQNMGENKAAEIKDFQIVVADKGEGKKQQLAPKRSSNKDRHTKVEGRGRRIRMPALCAARIFQLTRELGHKSDGETIQWLLQQAEPSIIAATGSGTIPASALAAAGGSVSQPGASLSAGLHQKMEDLGGSSIGSGSSRTSWTMVGGNLGRPHHVATGLWPPVSGFGFQSSSGPSTTNLGSDSSNYLQKLGFPGFDLPASNMGQISFTSILGGANQQLPGLELGLSQDGHIGVLNPHALNQIYQQMEQARMQPQHQHQHQQQPPAKDDSQGSGQ

>Cotton_D_gene_10008880

MIMDGENGISTSKFPLQLLEKKQPPCPNKGNSETSSEPPKKTPPKRTSTKDRHTKVEGRGRRIRMPATCAARVFQLTRELGHKSDGETIEWLLQQAEPAVIAATGTGTIPANFTSLNISLRSSGSTLSASHLRNTYFNPNFTSQQLRNMADDSSSHQQRRILFPEDSLNFPNVNTFLQTKQEMRDSTSVDFSAAEDTNKGKKRRPEHHELSQNQVGNYLIQSTMGSVPATFWTITNPSNQGISGSGAADPMWTFPSANNTNMYTSTTSSGVHFMNFASPMSLLPGQQLGTGIGAGGSFSDSHLGMLQALNAYRPTHSTNVSESPATGSHQVLHHGEEHRHDSSS

>Cotton_D_gene_10010801

MGESKTAETKDVVVADKEEGKKQQLAPKRSSNKDRHIKVEGRGRRIRMPALCAARIFQLTRELGHKSDGETIQWLLQQAEPSIIAATGSGTIPASALAAAGASVSQQGGSLSAGLHQKMEDLGGSSVGSGSSRTNWGMVGGNLGRPPHVAAGSWPPVSGYGFQSSSAPSTTNLGSEGSNYLQKIGFPGFDLTATTMGQMSFTSMLGAANQQLPGLELGLSQEGHFGVLNPQALTQIYQQMGQARVHQQQQQQNHHHHHHHHNQPSAKDDSEGSGE

>Cotton_D_gene_10012543

MECNRNQTIEEIDESINNNTAVIINEERHQSNNTTPTFSASDATVMDPNQPMKEEALTDTEPQELANPTLVQVVPAARTQRSLAPKRPSKDRHTKVEGRGRRIRMPAACAARIFQLTRELGHKSDGETIRWLLEHAEPAIIEATGTGTVPAIAVSVNGTLKIPTSSGKQEGELPKKRRRRPSNSEFIDVNEHQSSVSSGLAPIAPMTCNSFNLNSQGLVPIWPMGTFSVLPAAPGSNQAQLWAIPATATPFFNVTGRPISSFVSAMQPEVQDSSVGSMGSSVLPTSSSGATSVSNGNNSSNHSTTQMLREFSLEILDKRELQFLGRPANQQAPCSKP

>Cotton_D_gene_10016293

MESQSNKSSNSSEHHHHHHGQKQEASLQLVSRDGQPQAAAGAAHGTSSHQAQAQTQAQPQAPGHHVSFMGSISNQIGVPSSSPTSTSSLAKPPAAKRPTKDRHTKVDGRGRRIRMPAVCAARVFQLTRELGHKSDGETIEWLLQQAEPAIIAATGTGTIPANFSTLNVSLRSSGSTISAPSSKSAPLSFHSALGFYNSNGDEARRIGNSTAMLGFHHQLYPQLLHPETHMRSGSNPNDNYATKPFRDDLFKETSQHNAETGAIDANSPKPERTGMPEQEPGLFQTANVMPAPAMWAVAPATTNGGNAFWMLPVGGGATAASATVPEAQMWTFPAHYSGGGRGNPVQLGSMILQQQQAGGQQLGLGVTETNMGLLGSGMNVYSNNNRVGLKMNLEQQHHHENQTQGSDSGDENPATDSQ

>Cotton_D_gene_10016960

MDPSEDGGTSDLSTSTGGPNNATPTAANSIKNDENCENSALMVMPLKEEPIEPDPDRKTHPPRPTRVVPVSGQMSMQIPIPTPVTTAKRSYNKDRHTKVEGRGRRIRIPATCAARIFQLTRELGHKSDGETVRWLLEHAEHAIIEATGTGTVPAIAVSVGGTLKIPTTSATNTSNNNSQIDDKTKKRKRPASSEFCDVNDGIPIAVAQQQQQQQRLVTQYSGLAPVAQQALVPVWAVGNTGMMVPANAFWMIPQSTPTASGDWLTNQRPPPQLWAFSPALTSVCNIAARPTSSFVATTTETQAVVCDGVSTLAVSTSSTPVGATEAKKSTMAPSVSSGGNGGGGTGGTAQMLRDFSLEIYDKQELQLMGRSWNHHQQQIKAASKQKNP

>Cotton_D_gene_10017148

MDLDDDGGTSDLSTSAGEPNAATDKICESSAFQIKPFKEEPIDSHPPPPIGVVPVSMRPLPTVKRSSTKDRHTKVEGRGRRIRIPATSAARIFQLTRELGHKSDGETVRWLLEHAEQAIIEATGTGTVPAIAVSVGGTLKIPTTAGSNASNNKNNNNQIDDDGGATKKTKRPAKSEFCDINDGNPFAISNQQQLVTQASGLAPVTPQGLVPVWAVGNTGMMIPANSFWMIPQPTATGAANGRLSNQQPSSPQIWALSPSVFNVAARPISSIVSTSNQSVCNGLSTSAVNRSSTAVAKKSTMAPSGSSSGGKAQMLRDFSLEIYDKQELQLMGRSESHQAADASCF

>Cotton_D_gene_10017874

MFPSNSNGNNNDPITYLDHSILPLSFFHSPSSPNYNQCELLQLDEYDYDVLWNQQQHFDDDDDQFLHQTTLLTDNSVSETIVNLPDCRHNNTTTDIHQQPMPRKRPAASKTDRHSKINTANGPRDRRMRLSLDVAREFFGLQDMLGYDKASRTVEWLLVQAKPEITKLMNNNNNSFGFAKSPSSTSETEVVSGIDKAAAIDGNIPKGTPSKKEKKERRQRKTSFRPLARDMRVKARERAKARTKEKNMSLRLNNETRDNDPNRFSSSWSSTWTKQPGIQNHHNNNNTVFQADNINIHGDRMIWSLNCLQNTGLINQEVSHKCPLSQIYIYISNMCIPLINLYICLQLTGSILW

>Cotton_D_gene_10022676

MEGGGSDDHQLHHHHQHRPTFPFQLLEKKQDDNQPCSSSSSPPFPSLLPPSSSSSPSADQPNTSRSISSLQISPEPCKKPPPKRTSTKDRHTKVDGRGRRIRMPALCAARVFQLTRELGHKSDGETIEWLLQQAEPAVVAATGTGTIPANFTSLNISLRSSGSTMSVPSQLRSSGFNSNFSMQQRRSLFPGIGLETTPTFLNFQSSSNLNSMFQAKQELRDHNSSSLEISANEENSLGRKRRPEQDLSSQHQMGSYLLQSSTGVIPAATHGQIPANFWMVTNSNNQVISGDPIWTFPSVNSSALYRGTMSSGLHFMNFPAPMALLPGQQLGSSGVTSGAAGGGGGGGSTGISAEGHLNILAGLNPYRQVSESQASGPHSNNDGGGGGGAGGGGSAGGGDNRHDATSHQS

>Cotton_D_gene_10025316

MASSKNMGLEMALSTNPSTKKPRTGSANKASTSKNKKTTTKDRHAKVDGRDRRIRLPTVCAARIFQLTRELGLKTDGETIAWLLRQAEPSIVAATGTGVSETSTTPGPTNNNSTNTGLVPFSTGSDSPLATSDYTPCLTFLQEPDRMLLKNSGDVSRGEPSLPPLEFDFDLEFSAHEMAMFQSVAAAANHDKEGKI

>Cotton_D_gene_10027048

MISSSKNTDFPTRQDEDDDKRNETNKGPSSSSSSSQWLRLKDPRIVRVSRAFGGKDRHSKVYTIRGLRDRRVRLSVPTAIQLYDLQDRLGLNQPSKVVDWLLNAAKHEIDELPPLPMPPPPANFPISDAFWRTKSKEVTRDVIDDKDDKHRRNEGDDDDDHGTQHVSSGSYYHFEPSNFPLSSHLVSHGFAVHAPQVDNFNVVAAPLPSALSLCPLPPPQPGGGAPPLFPPHVVDPRQQVNHFQMLSSGAQQNLLLNSLNFPPITQSFRPFQLMPPRFPHNQPDK

>Cotton_D_gene_10028625

MDPKGSKQPPAEVSNFLSLPPQPQQQPHQSMGENKAGESKGFRIVASKEEGKKQQLAPKRSSNKDRHTKVEGRGRRIRMPALCAARIFQLTRELGHKSDGETIQWLLQQAEPSIIAATGSGTIPASALAAAGGSVSHQGASLSAGLHQKMEDLEGSSIGSGSSRTSWATVGANNLGRPHHVATGLWPSVSGYGFQTSSGPSTTNLGTESSIYLQKIGIPGFDLPATNIGQMSFTSILGAANQQLPGLELGLSQDGHIGVLNHQALSQIYQQMGQARVLQQQQNQQNPPAKDDSQGSGH

>Cotton_D_gene_10029652

MSNSETAATNGSLMDPQRHQAPAANGSLAVKKPPTKDRHSKVDGRGRRIRMPIICAARVFQLTRELGHKSDGQTIEWLLRQAEPSIIAATGTGTTPASFSTVSVSLRGANSTSLSSTTSSSLDHKPLLGPTPFILGKRVRSDDDNAGKDDSGGLTVGPGVGSIVGPNGTHPGGFWALPARPDFGQLWSFAAPPPPDMVVQAAAQQPAAAAALFVQQQQAMGEASAARVGNYLPGHLNLLASLSGAPGGSGRRDEDPH

>Cotton_D_gene_10029958

MISSSREAKQEGETNDGSGGGNKLSKGPSSSSSSRHWSSAGFRNPRIVRVSRSFGGKDRHSKVCTVRGLRDRRIRLSVPTAIQLYDLQERLGVGQPSKVVDWLLEATKDDIDKLPPLQMPLGFNNQFHQPFLVPHEPNPSFLDPNSMLMKDGEEEDQRMAGDRDKGKWIKMNEDENHGGNNNNNNIEDQRLFPLTNHSPFPGLLNNGMPLNSCYHWEPSGLSLSQFGNHGLMAPQTENFFNGNTTSVPLPCSTTVPSATMASFFPTYTPYGTNPGSNDSRQMNYLQLLSSNFLSNSMKPFSLNVNAGLTHTQNDDENHGDDQDNTDS

>Cotton_D_gene_10030066

MSNSEAAAKNGATMDTQRQQAPGNGALAVKKPPSKDRHSKVDGRGRRIRMPIICAARVFQLTRELGHKSDGQTIEWLLRQAEPSIIAATGTGTTPASFSTVSVSVRGGANSTSLPSTTSSSLDHKPLLGSTPFLLGKRVRPDDDNAGKDDSGGATVGPGVGSIVGPAGTHTGAFWAVPARPDFGQIWSFAPPPPPEMVVQTAAQQPAAAAFFVQQQQAMGEASAARVGNYLPGHLNLLASLSGAPGGSGQRDDDPR

>Cotton_D_gene_10030346

MFFSTSANDYNNINPFLHFPSSSYHPQALPPPPPPPLLSHESNDILLNHHHHHHDLVSASSLLPANPQLTDTLLNMALLNKDGVGFGGPPGFGFPVNKAVKKDRHSKICTAQGVRDRRVRLSIEIAREFFDLQDMLGFDKASKTVEWLLRKSNNAIRELVKMKRYGNGCPGGQRSFSLVPDDQYEMVAENGALGVDGGEFEGTAFQSNLLELEGVVSKDKKMKILHKAAVPLLAKESRAKARARARERTREKMCSGSGSSTSRHEWKICPDSSPHFLRSLSQLEPTKKSDHSYGHNSSSMASSSKVVAHQVEEPSAASRDNVIEESLVIRRMLKPSAILGFQQNLATSKDASCNSSGNNGFPNLSQKWDINGAMAHSTLCAVTTNVSLSTGVQLYGKP

>Cotton_D_gene_10030663

MYPSNSNGSSEEPINDHSFFHFVMDSKQEDPSFGFFNFPSPYYSQCELEQVFQDQDHDVFLHQHHDLLFHHEQNHQPLMADSVSGTIVNVPATSAVDCTKKKSNNHNADTEVKQQKRSSSKRDRHSKINTANGPRDRRMRLSLDVAREFFGLQDMLGYDKASRTVEWLLIQARHEIMKLARTRVSHNNINSVAAAAAAEAAAVAKSPSSTSEGEVVSALEGTISKGKPTEKTVKKTTFQFHPLARDLREKARARAKARTKAKNMWNQKLCCSTDETKLSSWWSFQTGEESSIQQHHHNKKINPSLQQVAGGDMIDESLAIINKWNPDSMFNCLQNSGINQEHQITGFLPFGKPWEGYNNKI

>Cotton_D_gene_10031549

MEVDEIQTQTCKFSTVGNGRNETSKIGQKGSDSNFPDDEEDGELIKRAAATAVANGGVADTIGTNSLRGWHNSSRIIRVSRASGGKDRHSKVWTSKGLRDRRVRLSVTTAIQFYDLQDRLGYDQPSKAVEWLIKAASDAIAELPSLNTSFPDTPKQLSDEKRGSGGIEQGFDSAEVELDGDPSNYQQHLSLSKSASSSTSETRKNSSLSLSRSGTRVNRVKARERARERTAKEKEGHQQQNVSPISQNSSFTELLTSGIGNVSNNNTSPSASASASAHQDPSAESDFYQKANSTARLWPVTPMDYFASGLLGPSSSRGHHSSGFPGGQIHLVNSLQQPMTTPPFTVSGENHQEMQHFSFVPNPDHLIPVATTQPGQGVDYNLNFTISSGLAGFNRGTLQSNSPFLPPHLQRFSSIDGSSPLYIGTPPVENHHHHQFTAGLDGRLQLCYGDGNRSSDQKGKGKN

>Cotton_D_gene_10033147

MEGGGSDDHHHHHHSHHHHHQQPLHHHHHHHHRPTFPFQLLEKKEEDNQPCSSSSSLPFPSLPVSSSSADQHTSTRSISTHQISPETSKTAPPKRTSTKDRHTKVDGRGRRIRMPALCAARVFQLTRELGHKYDGETIEWLLQQAEPAVIAATGTGTIPANFTSLNISLRSSGSSMSVPSQLRSSGFSSNFSMQQRRSLFPGIGLETTPTPTTFLNFQSSSTSLGISEEAEESSLGRKRRPEQDLSSQHHQMGSYLLQSSTGTIPASHHGQVPANFWMVTNSNNQVMSGGDPIWTFPSVNNSGLYRGTMSSGLHFMNFPTPMALLPGQQLGSSSSGADGGGGGGGSGGSSGISEGHLNMLAGLNPYRQDSGGTGVSESQVSGSHSHHGGGGGSDE

>Cotton_D_gene_10033515

MIMDENGIRRPNFPLQLLEKKEQPRSDPADVDTSSKPTLEPPSKKPPPKRTSTKDRHTKVEGRGRRIRMPATCAARVFQLTRELGHKSDGETIEWLLQQAEPAVFAATGTGTIPANFTSLNISLRSSGSSMSASYLRNTYFNPNFTTQKLRTGSEWDQRNVLDDSSQQQQQQRRVLFPGVALSSEDSLTFPGTSSTTLNAFLQAKQEIRDAESADTSIGKRRRPEQQESPQNQVGSYLIQSNTGSIPTSHNPIPATFWIGNQVISGSGTGDPMWTFPSTNNTNMYRGTMSSGVHFMNLAAPMALLPAQQFGSGISAGGGSVTDTHLSMLASLNAYRPVPGIGVSEPPAGGSH

>Cotton_D_gene_10033516

MIMDENGIRRPNFPLQLLEKKEQPRSDPADVDTSSKPTLEPPSKKPPPKRTSTKDRHTKVEGRGRRIRMPATCAARVFQLTRELGHKSDGETIEWLLQQAEPAVFAATGTGTIPANFTSLNISLRSSGSSMSASHLRNTYFNPNFTTQQLRTRSEWDQRNVLDDSSQQQQQQRRVLFPGVALSSEDSLTFPGTSSTTLNAFLQAKQEIRDAEAADTSIGKRRRPEQQESPQNQVGSYLIQSNTGSIPTSHNPIPATFWIGNQVISGSGTGDPMWTFPSTNNTNMYRGTMSSGVHFMNLAAPMALLPAQQFGSGIGAGGSSVTDTHLSMLASLNAYRPVPGIGVSEPPAGGSHQHHGGENRHDSTS

>Cotton_D_gene_10033598

MEIDEIQTQQGSKFSRVGNGRSESSRMGQKGSDNYYPDDEEGREVMKRASANGGGGDALADTAAANRLRGWHHSSRIIRVSRASGGKDRHSKVWTSKGLRDRRVRLSVTTAIQFYDLQDRLGYDQPSKAIEWLIKAAADAIAELPSLNTSFPDTPRQLSDDGTEQGFDSAEVELDGDPNNYQENQSQQHLSLSKSACSSTSEISKNSGLSLSRSENRVKARERARGRVAKEKGKEQKTDIAHQQNVNPISHNSSFTELLTCGIGSVSNNHTSPSPTASARQNPRQWPVTQMDYFTMGLLGPSSSRNQSSGFPGQMQQPQPILIPPFTVSGENNQKLQHFSFVPNTDHMIPVATAQPVLGSDYNLNFAISSGIAGFNRGTLQSNSPPFLPHHLQRFSSIEGSPPVENHHHH

>Cotton_D_gene_10034674

MSNSEGGANNGAIMDPQRQQATVNGSLAIKKPPSKDRHSKVDGRGRRIRMPIICAARVFQLTRELGHKSDGQTIEWLLRQAEPSIIAATGTGTTPASFSTMSVSVRGGANSTSLSSTTSSPSLDHKPLLGPTPFILGKRVRSDDDNPGKYDTGGVMVGPGVVGPAGGFWALPGRTDFGQLWSFPPPAEMAAHQPAAALFVQQQQAMGEASAARVGNYLPGHLNLLASLSGAPGSSGRRGEDPR

>Cotton_D_gene_10034792

MEPKGKGKGKGKGSNHHPQEVPTCLTPQKAENNKPAEIKNLQIMIASKDDNKKQLAPKRSSNKDRHKKVDGRGRRIRMPALCAARIFQLTRELGHKSDGETIQWLLQQSEPSIIAATGTGTIPASALAAAGASVCAQGNSVSAGLHTKMGLGACTGSKDRNNWAMLGGNLGRSQIPSGAWSSSNGIGSGLVQVSEQSTSASNFGNENSNHIHHNYGFQGLEFPNMNMGFVSFSSLLNGSNLQVPGLELGLSQDPHFGVSNSQAFSHFYQQIGQQRGGVRPLNQQQIVADKDNSQGSKQ

>Cotton_D_gene_10035275

MDSQTQSVDQQQQEEENEQRPFLPENAEEEGGPTSLQPSKKRRRYKGDRHIKVDGRDRRIRIPLTCCSGLFRLTREMGHRTNGETIQWLLQQTRPDLVPPDPVTHPTLLFASAPAYMEKGRVRATVVQASTVFFDTPATLDKAERLIAGSAAYGSQLVVFPEAFVGGYPRGFPFESPNEDNQELPKYHASAIEVPGPEVDRLAKISCRYKVHLVMGVVEKDGFYLFSTILFFDPVGRYLGKHRKLMRSASECVVWCSGEKSSLPLYRTAIGKVGGLLYLDNRIPSLRTELYAKGIQMYCAPTADAREEWRASMIHIAIEGRCFVLSANQFCRRKDYSLPLKCIDGDSNSDLLDTIVCSGGSVIVSPSGTILAGPNYQGESLISADLDLEEITRAKLEFGEVGLGMGPDSVGWSANKPNLVLYQTAVKTEAFVDLS

>Cotton_D_gene_10039906

MGESNHQAATSSRLGLKHSGGEIVEVQGGHIVRSIGRKDRHSKVCTAKGPRDRRVRLSAHTAIQFYDVQDRLGYDRPSKAVDWLIKKAKSAIDELAELPPWNPQTLTTTTSTTKQNNQQNQNIITAVDNEKPRRTATLMGNQVQILQQQGTGDNPNSNSGFLPSSLVSDEIADTMKSSFPLGASSEAPSSSIQFQNYPPDFLSKTSSHSQDLRLSLQSFPEPVLLHHHHQAAAAQAHQTESVLFSAGTSPLAGFDGSSAGWENHHHHPAEVGRLQRLVAWNNGAAAADTGSGGGGGMGGFLFGNLSAPPLSPAFGQNGQFFFQRGPLQSSNTPLVRAWIDQPIPTTDEYHHHHHQIPQNIHHQPALSGIEFTTSGVFSGFRVPARFQGAQEDQDSIANKLSSASSDSHH
